# Cataract of the Eye

**Published:** 1883-04

**Authors:** 


### CATARACT OF THE EYE.

The front portion of the eye is filled with a transparent, watery fluid called the aqueous; the large back part of the eye with a transparent gelatinous fluid, and is called the vitreous. Between the two is the crystalline lens, by which, mainly, the rays of light that enter the eye are centred upon a thin membrane, called the retina, there forming the minute image of everything seen.

This crystalline lens is liable to become more or less cloudy, thus wholly, or partially, preventing the passage of light through it. This is cataract.

The opacity—or inability to allow light to pass through it—may be in the nucleus, or central portion, or may, for a time at least, be in the outer portion, called the *cortex*. It is sometimes caused by blows, sometimes by inflammation extending to it from other parts of the eye; but in most cases it is impossible to detect any exciting cause.

One form of it tends to develop mainly somewhat late in life. The opacity may increase very slowly, or at a more rapid rate, but still gradually; or slowly for a long time, and then with great rapidity, ending within a few days in total blindness. A cataract in one eye may be expected sooner or later to manifest itself in the other.

Many persons allow themselves to be blind for the rest of their lives, not knowing that good, serviceable sight might probably be theirs. By the improved methods of the present day, the oculist succeeds in restoring the sight in nine cases out of ten, the final success depending on the patient's general health, favorable surroundings and the faithfulness with which instructions are followed during subsequent treatment. No matter how old the person is, provided his health and his eyes in other respects are in good condition.

It is important that the opacity be brought to the oculist's attention early.—*Youth's Companion.*

---

THE migration of birds seems to be more a question of food than anything else. In the "Proceedings of the Philadelphia Academy of Natural Sciences," just issued, note is made of the migration of the robin during July and August of the past year. The extraordinary dry season seemed to have shortened the supply of food, and they were noted in immense numbers traveling from many miles toward the swamps of New Jersey, where berries abounded.

